# Low-dose CT combined mammography in diagnosis of overflow breast disease: A protocol of systematic review

**DOI:** 10.1097/MD.0000000000021063

**Published:** 2020-07-02

**Authors:** Hao Tian, Shao-jun Hu, Qun Tang, Fei-hong Ma, Rong-rong Yao

**Affiliations:** a Department of General Surgery; b Department of Tumor Surgery; c Department of Hematology; d Department of Interventional Radiology, First Affiliated Hospital of Jiamusi University, Jiamusi, China.

**Keywords:** computed tomography, diagnosis, mammography, overflow breast disease

## Abstract

**Background:**

Overflow breast disease (OBD), also known as breast nipple discharge, refers fluid or liquid that comes out of nipple. Many patients with breast cancer experience such condition. However, it is not easy to detect it at early stage, especially for pathological OBD. Previous study found low-dose CT combined mammography (LDCTMG) could help in diagnosis of OBD. However, there is no systematic review investigating this issue. Therefore, this study will examine the accuracy of LDCTMG in diagnosis of OBD.

**Methods:**

This study protocol will search literature sources in electronic databases and other sources. The electronic databases will be retrieved in The Cochrane Library, the Cochrane Register of Diagnostic Test Accuracy Studies, PUBMED, EMBASE, Web of Science, CINAHL, CNKI, and WANGFANG from inception to the present. We will also search other sources. All literature sources will be sought without restrictions to the language and publication status. Two researchers will independently carry out study selection, data extraction, and study quality assessment. Statistical analysis will be performed using RevMan 5.3.

**Results:**

This study will exert a high-quality synthesis of eligible studies on the analysis of LDCTMG in diagnosis of OBD.

**Conclusions:**

The results of this study may provide evidence to help judge whether LDCTMG is accurate in diagnosis of OBD.

**Study registration:**

INPLASY202050116.

## 1 Introduction

Overflow breast disease (OBD), also known as breast nipple discharge, is defined as any fluid or liquid that comes out of nipple.^[1–3]^ It is 1 of the most common complaints in breast disease,^[4–6]^ and consists of physiological OBD and pathological OBD.^[7–9]^ Of those, physiological OBD refers to discharge during the period of pregnancy and lactation, or discharge in postmenopausal women, or resulting from oral contraceptives or sedatives.^[3,10]^ Pathological OBD refers intermittent or continuous discharges from 1 or both sides of breasts from months to years under non-physiological condition.^[11–13]^ Of these, pathological OBD accounts for 2.3% to 17.5% of all breast diseases, and about 1/15 patients with breast cancer experience such disorder.^[14–15]^ However, it is till difficult to diagnose pathological OBD at early stage. Fortunately, studies have reported that low-dose CT combined mammography (LDCTMG) can help diagnose such condition.^[16–22]^ Currently, no systematic review has been conducted to assess the accuracy of LDCTMG in diagnosis of OBD. Thus, this study will aim to explore its accuracy in diagnosis of OBD systematically and comprehensively.

## 2 Methods

### 2.1 Study registration

This study was registered through INPLASY202050116. We organize this study based on the Preferred Reporting Items for Systematic Reviews and Meta-Analyses Protocols guideline.^[23]^

### 2.2 Inclusion criteria for study selection

#### 2.2.1 Types of studies

We will include case-controlled studies on evaluating the accuracy of LDCTMG in diagnosis of OBD. However, other studies will be excluded, such as review, comments, case studies, non-clinical study, and uncontrolled study.

#### 2.2.2 Types of participants

All female adult participants who were diagnosed as OBD will be included in this study, in spite of country, race, and different characteristics of OBD.

#### 2.2.3 Types of tests

Index test: All participants received any forms of LDCTMG in diagnosis of OBD.

Reference test: All subjects received approved histopathological test in diagnosis of OBD as a comparator.

#### 2.2.4 Types of outcome measurements

Outcome measurements comprise of sensitivity, specificity, precision, accuracy, false positive rate, true positive rate, false negative rate, true negative rate, and diagnostic odds ratio.

### 2.3 Search methods for study selection

#### 2.3.1 Electronic databases

We will perform primary literature search from inception to the present in The Cochrane Library, the Cochrane Register of Diagnostic Test Accuracy Studies, PUBMED, EMBASE, Web of Science, CINAHL, CNKI, and WANGFANG. We will not place restrictions to the language and publication status. We will build detailed search strategy of PUBMED in Table 1. We will adapt similar search strategies for other electronic databases.

**Table 1:**
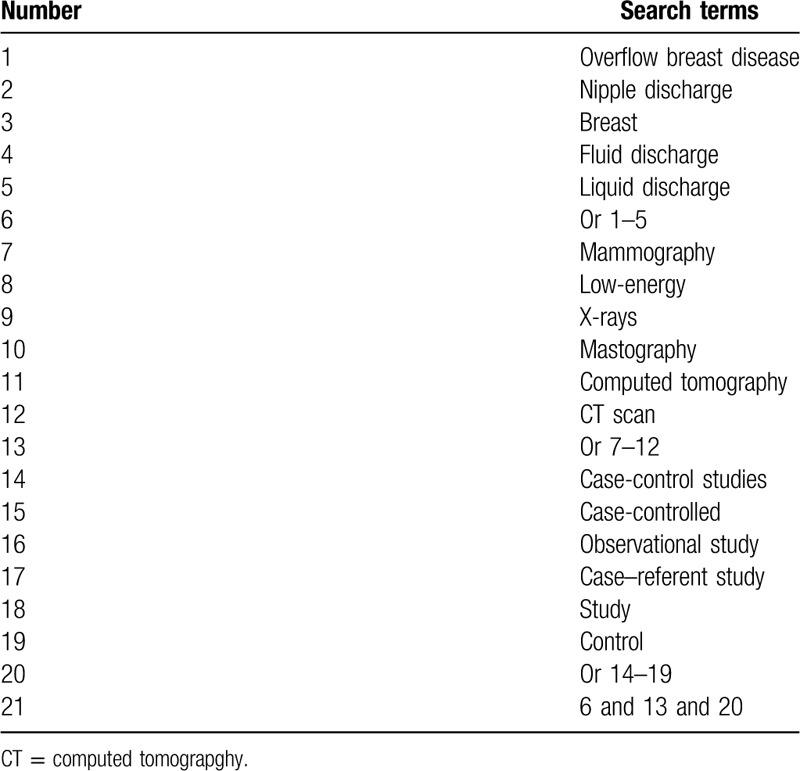
Search strategy of PUBMED.

#### 2.3.2 Other resources

We will carry out secondary literature search from other sources, such as conference proceedings, ongoing studies from clinical study registry, and reference lists of included study.

### 2.4 Study selection and data extraction

#### 2.4.1 Study selection

Two researchers will independently select studies based on the eligibility criteria. This study will include a 2-step process. At the first step, we will focus on scanning titles/abstracts of searched studies to eliminate irrelevant studies. At the second step, we will read full-text of potential studies cautiously. Any doubt will be solved by a third researcher through discussion. The process of study selection will be presented in a flow chart.

#### 2.4.2 Data collection and management

Two researchers will develop a data collection sheet and will collect data from eligible studies. The following data will be collected, including study characteristics (title, first author, time of publication, country, et al), patient characteristics (race, age, diagnostic criteria, eligibility criteria, et al), study setting, study methods, sample size, study quality, index and reference tests, outcome indicators, results, findings, and conflict of interest. We will solve disagreements by a third researcher via discussion or consultation.

#### 2.4.3 Dealing with missing data

We will contact primary study author to request missing or unclear data by email or telephone, or fax. If we can not obtain such data, we will analyze data using intention-to-treat analysis.

#### 2.4.4 Risk of bias assessment

Two researchers will independently assess risk of bias for included study by a Revised Tool for the Quality Assessment of Diagnostic Accuracy Studies.^[24]^ Any doubt between 2 researchers will be cleared up by a third researcher through consultation.

### 2.5 Statistical analysis

#### 2.5.1 Data synthesis

We will perform data analysis using RevMan V.5.3 software and Stata V.12.0 software. For each eligible study, we will present outcome indicators as separate binary classifiers and record specifics for dichotomization. To visualize outcome results, we will present them as descriptive statistics and 95% confidence intervals. We will carry out *I*^*2*^ test to check heterogeneity across studies. *I*^*2*^ ≤ 50% means minor heterogeneity, and Mantel-Haenszel fixed-effects model will be used. On the other hand, *I*^*2*^ > 50% indicates substantial heterogeneity, and Mantel-Haenszel random-effects model will be placed. We will construct 2 x 2 tables to estimate reference standard and test outcome, and will conduct a descriptive forest plot and a summary receiver operating characteristic plot. If necessary, we will carry out meta-analysis based on sufficient similarity in characteristics of study and patient, index and reference tests, and outcome indicators. If meta-analysis is deemed not to be conducted, we will report study results by a narrative description.

#### 2.5.2 Subgroup analysis

This study will carry out a subgroup analysis based on the different patient characteristics, index and reference tests, and outcome indicators.

#### 2.5.3 Sensitivity analysis

This study will perform a sensitivity analysis to examine the stability of study findings by excluding low quality study.

#### 2.5.4 Reporting bias

This study will undertake Funnel plot to check reporting bias if at least 10 eligible studies are included.^[25–26]^

### 2.6 Dissemination and ethics

This study will be published on a peer-reviewed journal or a conference meeting. No individual patient data and privacy will be obtained, thus, no ethic approval is required.

## 3 Discussion

The most widely recognized diagnostic for OBD is considered out of date and can not detect pathological OBD at early stage. Studies suggest that LDCTMG is utilized in diagnosis of OBD. However, there are inconsistent conclusions; and no systematic review focusing on this topic has been conducted. This study will summarize current study to systematically and comprehensively elaborate the accuracy of LDCTMG in diagnosis of OBD. The findings of the present study will yield evidence to judge the accuracy of LDCTMG for diagnosing OBD.

## Author contributions

**Conceptualization:** Hao Tian, Fei-hong Ma, Rong-rong Yao.

**Data curation:** Shao-jun Hu, Qun Tang, Fei-hong Ma, Rong-rong Yao.

**Formal analysis:** Hao Tian, Shao-jun Hu, Qun Tang, Rong-rong Yao.

**Investigation:** Rong-rong Yao.

**Methodology:** Hao Tian, Shao-jun Hu, Qun Tang, Fei-hong Ma.

**Project administration:** Rong-rong Yao.

**Resources:** Shao-jun Hu, Qun Tang.

**Software:** Hao Tian, Shao-jun Hu, Qun Tang.

**Supervision:** Rong-rong Yao.

**Validation:** Hao Tian, Shao-jun Hu, Qun Tang, Fei-hong Ma.

**Visualization:** Hao Tian, Shao-jun Hu, Fei-hong Ma, Rong-rong Yao.

**Writing – original draft:** Hao Tian, Qun Tang, Fei-hong Ma, Rong-rong Yao.

**Writing – review & editing:** Hao Tian, Qun Tang, Fei-hong Ma, Rong-rong Yao.
